# Supplementary material for: Mantle data imply a decline of oxidizable volcanic gases could have triggered the Great Oxidation
Source: Nat Commun. 2020 Jun 2;11:2774. doi: 10.1038/s41467-020-16493-1 (PMC7265485; doi:10.1038/s41467-020-16493-1)
Supplement: Supplementary file 1 — Supplementary Information [file 41467_2020_16493_MOESM1_ESM.pdf]

Supplementary Information for "Mantle data imply a decline of oxidizable volcanic gases could have triggered the Great Oxidation"

Shintaro Kadoya[1], David C. Catling[1],
Robert W. Nickal[2], Igor S. Puchtel[3] & Ariel D. Anbar[4]

1. Department of Earth and Space Sciences/ cross-campus Astrobiology Program, University of Washington
2. Geoscience Research Division, Scripps Institution of Oceanography
3. Department of Geology, University of Maryland
4. School of Earth and Space Exploration and School of Molecular Sciences, Arizona State University

Supplementary Figures

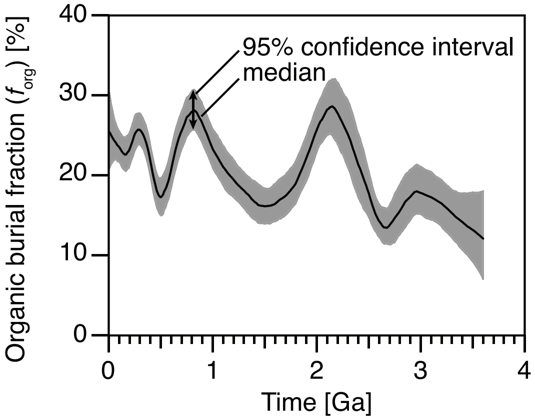

Supplementary Figure 1: Evolution of organic burial fraction (f_{org}) based on Krissansen-Totton et al. [1]. This diagram corresponds to the case of the locally weighted scatterolot smoothing (LOWESS) in Fig. 3C of Krissansen-Totton et al. [1]. The black solid line and gray shaded region represents the median value and 95% confidence interval, respectively.

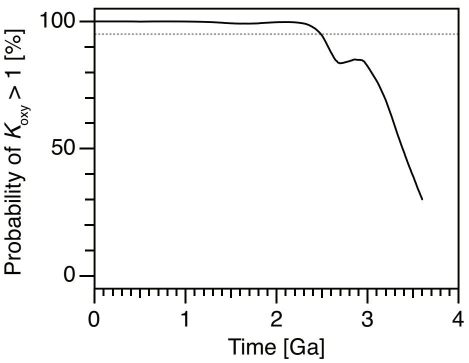

Supplementary Figure 2: Probability that the oxygenation parameter (K_{oxy}) is larger than unity ($P\left(K_{\mathrm{oxy}} > 1\right)$) as a function of time. The black solid line represents the $P\left(K_{\mathrm{oxy}} > 1\right)$ for the standard case (See also Fig.2b). The $P\left(K_{\mathrm{oxy}} > 1\right)$ tends to increase with time, which corresponds to the increase in K_{oxy} (See also Fig.2b). The $P\left(K_{\mathrm{oxy}} > 1\right)$ is 95% (the gray dotted line) at 2.4 Ga, which is the oxic transition time.

Supplementary Notes

Supplementary Note 1: Sensitivity test

In the main text, we assumed that the mantle temperature, degassing pressure, and the total flux ratio of carbon and sulfur (i.e., χ_{c} and χ_{s} in Equation 15), were constant. We also neglected the deposition of magnetite and the uncertainty of the oxygen fugacity of the modern mantle. Here, we examined the sensitivity of our results to changes in these parameters.

As a consequence of the sensitivity tests, we generally obtained trends consistent with previous works. As explained below, the mantle temperature has little effect on the oxic transition time (Supplementary Figure 3). On the other hand, a degassing pressure much higher than assumed in the the standard case can affect the oxic transition time (Supplementary Figure 5). The oxic transition time may also be sensitive to other parameters: factors related to sedimentary recycling of carbon and sulfur (χ_{c} and χ_{s}) as shown in Supplementary Figure 6, the deposition flux of magnetite (Supplementary Figure 7), and the oxygen fugacity that is assumed for the modern mantle (Supplementary Figure 8).

1.1 Mantle temperature

For our standard case (Fig. 2b), we assumed a mantle temperature of 1623.15 K, i.e., the modern potential temperature of the mantle [2]. In contrast, [3] estimated the modern redox proportions of volcanic gases using a temperature of 1473.15 K. However, it is likely that the potential temperature of the Archean mantle was higher today [e.g., 2, 4, 5]. On the other hand, the temperature for the gas vented from volcanic arcs would be lower than the mantle potential temperature because hydration associated with subduction would decrease the melt temperature, resulting in low degassing temperature. Hence, we calculated K_{oxy} with different temperatures (Supplementary Figure 3). In each calculation, we kept the mantle temperature constant and did not consider mantle cooling.

Supplementary Figure 3a shows the evolutions of $P\,(K_{\mathrm{oxy}} > 1)$ for different temperatures. As shown in Supplementary Figure 3a, higher temperature tends to result in higher $P\,(K_{\mathrm{oxy}} > 1)$, i.e., larger K_{oxy}. This is because an absolute value of oxygen fugacity of the mantle, f_{O2}, decreases with a decrease in the temperature. Here, note that the oxidation state of the mantle is represented as $\Delta\mathrm{FMQ} \equiv \log_{10} f_{\mathrm{O2}} - \log_{10} f_{\mathrm{O2,\,FMQ}}$, where $f_{\mathrm{O2,\,FMQ}}$ is the oxygen fugacity of the fayalite-magnetite-quartz buffer (see also Fig. 1). Since the $f_{\mathrm{O2,\,FMQ}}$ decreases with the decrease in temperature (Supplementary Figure 4), the f_{O2} of source melt, and therefore, f_{O2} of the volcanic gas do decrease with the cooling even if $\Delta\mathrm{FMQ}$ is constant. So, lower temperature results in more reducing volcanic gas (Supplementary Figure 3a).

Supplementary Figure 3b shows the oxic transition time for different temperatures. As explained above, cooling causes a decrease in K_{oxy} (Supplementary Figure 3a), the cooling also decreases the oxic transition time (Supplementary Figure 3a). However, such temperature dependence of volcanic gas is relatively weak and not significant for a mantle temperature exceeding 1400 K (Supplementary Figure 3a).

Given that cooling of the potential temperature of the mantle from 1900 K to 1600 K [e.g., 2, 4, 5], the hot mantle in the early Archean tends to cause a relatively high possibility of $K_{\mathrm{oxy}} > 1$, i.e., of the oxic atmosphere (Supplementary Figure 3a). However, the oxic transition time does not change so much (Supplementary Figure 3b).

On the other hand, arc volcanism seems to decrease the K_{oxy} (Supplementary Figure 3a) and the oxic transition time (Supplementary Figure 3b) considering that the hydrous phases within the oceanic crust lower the melt-

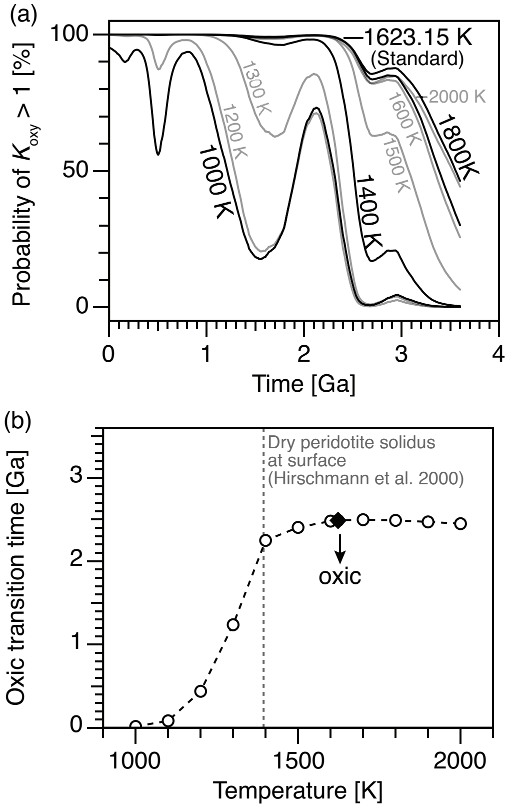

Supplementary Figure 3: A sensitivity study for mantle temperature: (a) probability that the oxygenation parameter is larger than unity ($P(K_{\mathrm{oxy}} > 1)$) as a function of time, and (b) an oxic transition time as a function of the mantle temperature. Here, the oxic transition time is the time at which $P(K_{\mathrm{oxy}} > 1)$ is 95%. Black diamonds represent outputs of the standard case (see also Supplementary Figure 2).

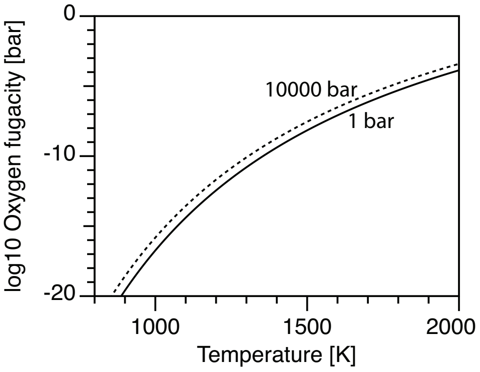

Supplementary Figure 4: Oxygen fugacity of the fayalite-magnetite-quartz buffer as a function of temperature. The $f_{\mathrm{O2,\,FMQ}}$ is calculated using a function given by [6].

ing temperature [e.g., 7]. However, we have to note that island arc basalts tends to be more oxidized than mid-ocean ridge basalt or oceanic island basalt [8]. Hence, to examine the oxidation effect of the volcanic gas vented from arc volcanoes, we also have to consider the difference in ΔFMQ between the mantle and the volcanic arc.

Comparison with previous study This trend of the oxic transition time with mantle temperature is consistent with [9] but inconsistent with [10]. The main difference of these studies is the assumption of the f_{O2} of volcanic gas. In [9] and this study, it is assumed that the f_{O2} of the volcanic gas is buffered by the f_{O2} of mantle. Hence, cooling results in a decrease in the absolute value of f_{O2} of the mantle and the volcanic gas, which makes volcanic gas more reducing.

In [10], it is assumed that the volcanic gas is a closed system, i.e., decoupled with its source melt. Under such a condition, cooling causes reduction of SO_2 to H_2S, which is accompanied by oxidation of H_2 to H_2O [10, 9]. Hence, the ratio of H_2O to H_2 in the volcanic gas increases with cooling, resulting in the apparent increase in f_{O2} of the volcanic gas relative to f_{O2} of the source melt. However, in a closed system, reduction of one gas must be accompanied by oxidation of another gas because the reduction of SO_2 is accompanied by the oxidation of H_2. Hence, the overall O_2 sink in the gas mixture does not change [9].

1.2 Degassing pressure

Volcanic gases tend to become more reducing with increasing pressure of degassing. Thus, previous studies [e.g., 11, 12] proposed that a change in volcanic degassing from submarine settings (where the weight of the ocean can add a few 100 bar) to subaerial settings triggered the GOE, though the effect of pressure might be overestimated [13]. Here, we calculated K_{oxy} with different degassing pressure (Supplementary Figure 3) and obtained a similar trend [e.g., 11, 12].

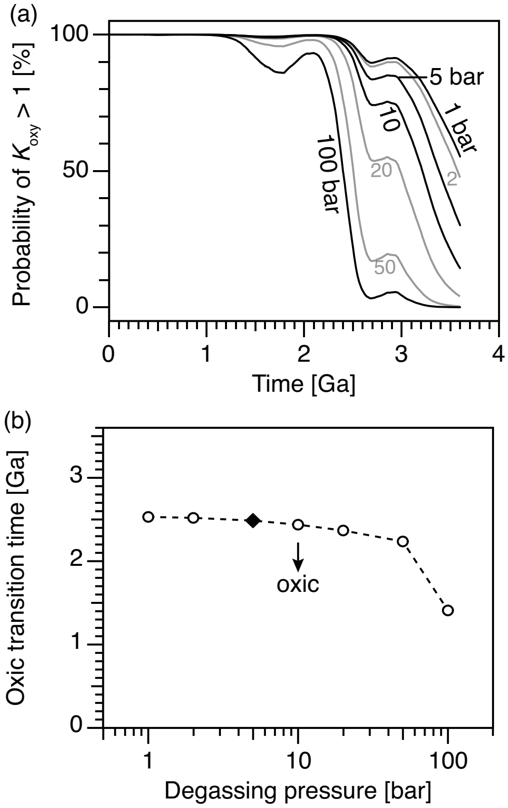

Supplementary Figure 5: A sensitivity study for degassing pressure: (a) $P(K_{\mathrm{oxy}} > 1)$ as a function of time, and (b) an oxic transition time as a function of the pressure. See also Supplementary Figure 3. The $P(K_{\mathrm{oxy}} > 1)$ tends to increase with the decrease in the mantle temperature (a), which is consistent with previous works [e.g. 11, 12].

Supplementary Figure 5a shows the evolutions of $P(K_{\mathrm{oxy}} > 1)$ for differ-

ent pressures of degassing. As shown in Supplementary Figure 5a, higher degassing pressure tends to result in lower $P\,(K_{\rm oxy} > 1)$, i.e., smaller $K_{\rm oxy}$. This trend is consistent with previous works [e.g., 11, 12]. Hence, the oxic transition time becomes smaller in units of Ga (Supplementary Figure 5b).

Thus, a transition from submarine to subaerial volcanism, i.e., a decrease in the degassing pressure, might have played a role in the oxygenation of atmosphere [11], in addition to secular mantle oxidation.

1.3 Total flux ratio of carbon and sulfur

We assumed constant total flux ratios of carbon ($\chi_{\rm c}$) and sulfur ($\chi_{\rm s}$) relative to total hydrogen in Eq. (2) for the standard case (Fig. 2), but they may have changed with time. Indeed, [14] assumed that the volcanic recycling of CO_2 and SO_2 increased with time as sedimentary reservoirs of carbon and sulfur grew and suggested that the ratio of CO_2/H_2O and SO_2/H_2O increased with time, which caused the GOE. Here, we examined the effect of ($\chi_{\rm c}$) and ($\chi_{\rm s}$) on $K_{\rm oxy}$ (Supplementary Figure 6) and obtained similar trend [14].

Supplementary Figure 6a and 6b show the evolution of $P\,(K_{\rm oxy} > 1)$ for (a) different ratios of a total carbon flux to a total hydrogen flux ($\chi_{\rm c}$) and (b) different ratios of a total sulfur flux to a total hydrogen flux ($\chi_{\rm s}$). As shown in Supplementary Figure 6a and 6b, higher $\chi_{\rm c}$ ($\chi_{\rm s}$) tends to result in higher $P\,(K_{\rm oxy} > 1)$, i.e., higher $K_{\rm oxy}$. This is because larger $\chi_{\rm c}$ ($\chi_{\rm s}$) tends to increase the numerator of Eq. (2). These results are consistent with the suggestion of Holland [14] that larger $\chi_{\rm c}$ ($\sim CO_2/H_2O$) and/or $\chi_{\rm S}$ ($\sim SO_2/H_2O$) tend to favor an oxic atmosphere and could drive the GOE.

Supplementary Figure 6c and 6d show the oxic transition time as a function of (c) $\chi_{\rm c}$ and (d) $\chi_{\rm s}$. Note that in Supplementary Figure 6c and 6d, black diamonds, which represent the oxic transition time of the standard case, are slightly offset from the dashed lines. This is because for the standard case, we integrated the oxic transition time using the distribution of $\chi_{\rm c}$ and $\chi_{\rm s}$. The increase in $\chi_{\rm c}$ ($\chi_{\rm s}$) increases the oxic transition time (Supplementary Figure 6c and 6d) because larger $\chi_{\rm c}$ ($\chi_{\rm s}$) results in larger $K_{\rm oxy}$ (Supplementary Figure 6a and 6b).

Estimating the evolution of parameters $\chi_{\rm c}$ and $\chi_{\rm s}$ is outside of the scope of this study but may be necessary for understanding of the evolution of Earth's surface oxidation.

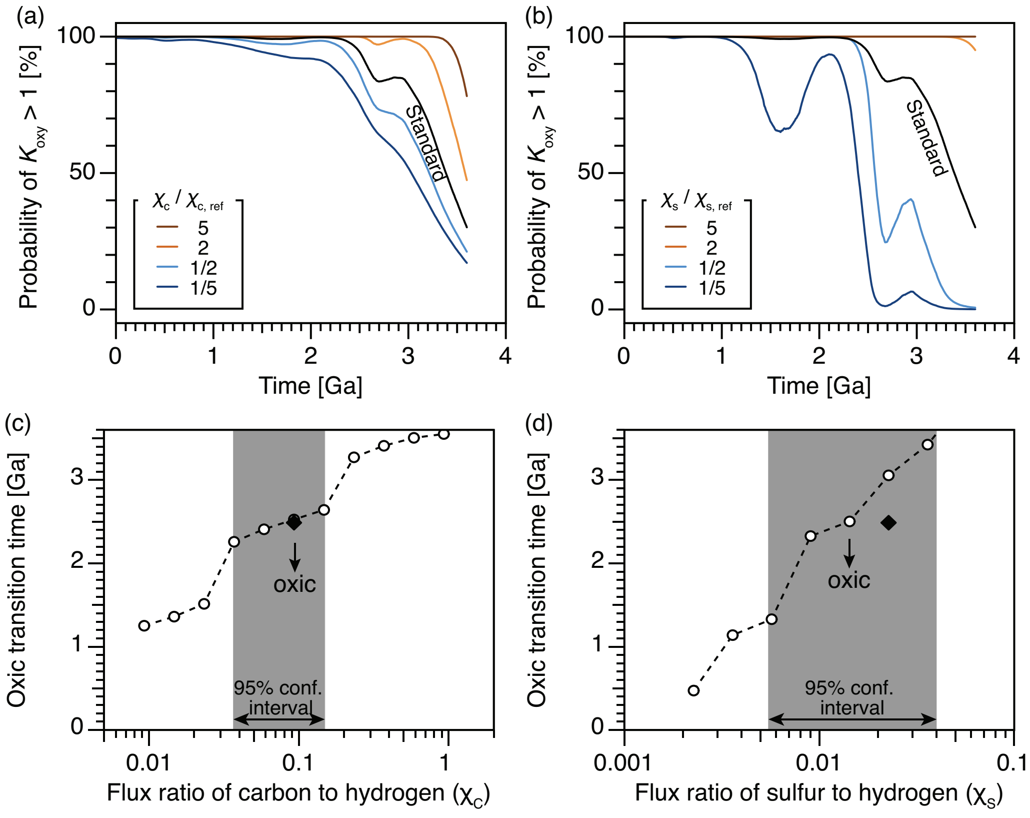

Supplementary Figure 6: A sensitivity study for the ratio of a total flux of an element (C or S) to the total flux of hydrogen: a and c are for the total carbon flux to the total hydrogen flux, χ_c, and b and d are for the ratio of the total flux of sulfur to the total flux of hydrogen, χ_s. The gray shaded regions represent the 95% confidence interval of modern values of χ_c (0.1 ± 0.06 [2SD]) and χ_s (0.023 ± 0.017 [2SD]), respectively. See also Supplementary Figure 3.

1.4 Magnetite deposition

The deposition of magnetite via serpentinization works as a sink of oxygen because it generates oxidizable hydrogen (simplified as $3FeO + H_2O \rightarrow Fe_3O_4 + H_2$), although serpentinization is a relatively minor sink today [15, 16]. However, in the Archean, serpentinization rates might have been enhanced by an oceanic crust including a larger amount of olivine. Such crust could have been produced by a higher mantle temperature because the higher mantle temperature would cause a greater degree of partial melting, resulting in an igneous product that is similar to its mantle source [17]. Hence, the deposition of magnetite might affect the redox state of the atmosphere on the Archean Earth [17]. Here, we calculated the K_{oxy} by varying the magnetite deposition flux (Supplementary Figure 7) and obtained a similar conclusion to Kasting [17].

Supplementary Figure 7a shows the evolutions of $P\left(K_{\mathrm{oxy}} > 1\right)$ for different fluxes of magnetite deposition. As shown in Supplementary Figure 7a, higher magnetite deposition flux results in lower $P\left(K_{\mathrm{oxy}} > 1\right)$, i.e., smaller K_{oxy}.

Supplementary Figure 7b shows the oxic transition time for different fluxes of magnetite deposition. The modern flux of magnetite deposition is estimated to be 0.05 to $\sim 0.2\ \mathrm{TmolO_2\,yr^{-1}}$ [15, 16], and the range is represented by the gray shaded region in Supplementary Figure 7a. Given the higher mantle potential temperature in the Archean, the oceanic crust would have been thicker and more olivine-rich than today [e.g., 18]. As a result, the flux of magnetite deposition may have been as much as 10 times or more higher than the modern flux ($\sim 2\,\mathrm{TmolO_2\,yr^{-1}}$) as suggested by [17].

1.5 Oxygen fugacity of the modern mantle

As explained in Supplementary Note 4, we anchor the evolution of oxygen fugacity (f_{O2}) of the mantle so that at $t = 0\,\mathrm{Ga}$ (now) the average value of f_{O2} is that inferred for the mantle from mid-ocean ridge basalt (MORB). Hence, the f_{O2} value chosen for the modern mantle anchors the absolute value of the trend of past f_{O2}, and therefore affects the calculated trend of K_{oxy} in the past.

For the standard case, we apply the average oxygen fugacity of the modern mantle, which is +0.2 in the $\log_{10}$ unit relative to the fayalite-magnetite-quartz (FMQ) buffer (i.e., $\Delta\mathrm{FMQ}_0 = +0.2$) according to [19]. However,

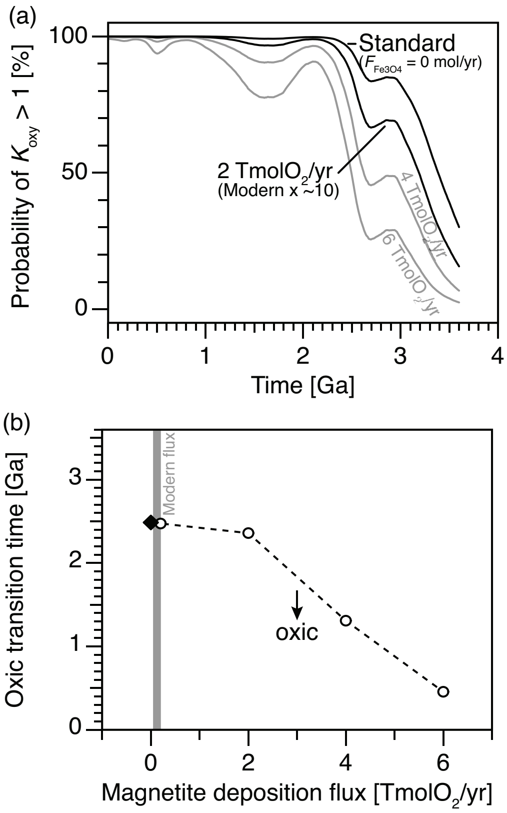

Supplementary Figure 7: A sensitivity study for deposition flux of magnetite (Fe_3O_4): (a) $P(K_{\rm oxy} > 1)$ as a function of time, and (b) an oxic transition time as a function of the deposition flux of magnetite. See also Supplementary Figure 3. The gray shaded region represents the range of modern flux of magnetite deposition [$0.05 \sim 0.2$ $\rm TmolO_2\,yr^{-1}$; 15, 16]. See also Supplementary Figure 3.

ΔFMQ_0 has an uncertainty of $\sigma = 0.3$ in $\log_{10}$ units [19]. Here, we investigated the effect of ΔFMQ_0 on the evolution of K_{oxy} (Supplementary Figure 8).

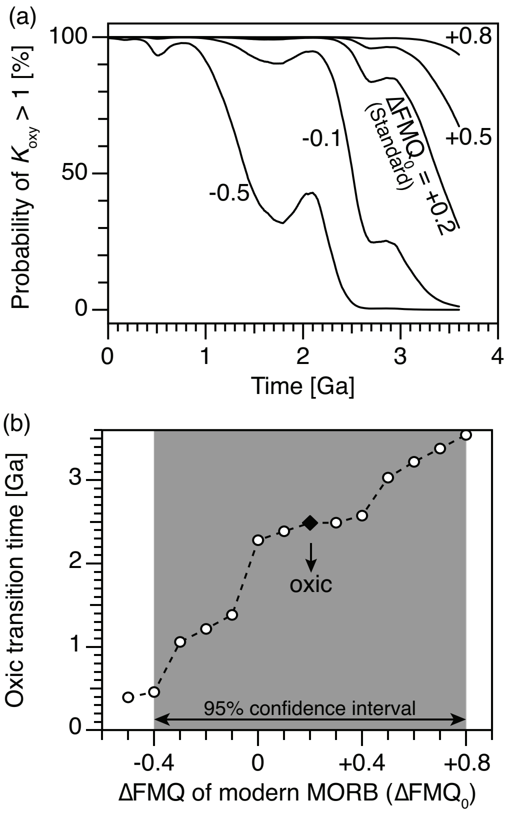

Supplementary Figure 8: A sensitivity study for the oxygen fugacity of modern mid-ocean ridge basalt: (a) $P\left(K_{oxy} > 1\right)$ as a function of time, and (b) an oxic transition time as a function of the pressure (See also Supplementary Figure 3) The gray shaded region represents the 95% confidence interval of the oxygen fugacity of modern MORB (FMQ+0.2 $\pm$ 0.6 [2SD]) according to [19]. See also Supplementary Figure 3.

Anchoring the f_{O2} trend to a large ΔFMQ_0 causes higher oxygen fugacity of the mantle throughout the past (Eq. (4)). So, this obviously results in a larger $P\left(K_{oxy} > 1\right)$ (Supplementary Figure 8a) and earlier oxic transition time Supplementary Figure 8b.

Supplementary Note 2: Holland's f number

2.1 Relation between Holland's f number and oxygenation parameter

As discussed in Kadoya et al. [9], the oxygenation parameter, K_{oxy}, of a closed gas mixture does not flip from < 1 to > 1 or vice versa if reactions occur within a closed gas mixture. Also, the same conclusion can be obtained in terms of Holland's f number. This point can be mathematically derived by considering the relation between f and K_{oxy}, as follows.

Holland's f number is calculated as follows [e.g., 20]:

$$\begin{aligned} f &= \frac{F_{\text{H2}} + (1 - 2f_{\text{org}})\,F_{\text{CO}} + (4 - 2f_{\text{org}})\,F_{\text{CH4}} - 2f_{\text{org}}F_{\text{CO2}} + 3F_{\text{H2S}}}{3.5F_{\text{S,all}}} + \frac{1}{3.5} \\ &= \frac{F_{\text{H2}} + F_{\text{CO}} + 4F_{\text{CH4}} - 2f_{\text{org}}F_{\text{C,all}} + 3F_{\text{H2S}}}{3.5F_{\text{S,all}}} + \frac{1}{3.5}, \end{aligned} \tag{S.1}$$

where F_x represents the flux of a material, x. Note that total carbon flux is $F_{\text{C,all}}(= F_{\text{CO2}} + F_{\text{CO}} + F_{\text{CH4}})$, and total sulfur flux is $F_{\text{S,all}}(= F_{\text{SO2}} + F_{\text{H2S}})$. The parameter, f_{org}, is the organic burial fraction, which was set at 0.2 in Holland [20]. In this formulation, we also consider methane flux, F_{CH4}, for consistency with the oxygenation parameter. Note that degassing of 1 mol CH_4 is equivalent to a source of 4 mol H_2: $CH_4 + 2H_2O \rightarrow CO_2 + 4H_2$.

The oxygenation parameter, K_{oxy}, is calculated as follows [e.g., 21]:

$$K_{\text{oxy}} = \frac{4f_{\text{org}} \times F_{\text{C, all}} + 5F_{\text{SO2}}}{2F_{\text{H2}} + 2F_{\text{CO}} + 8F_{\text{CH4}} + F_{\text{H2S}}}. \tag{S.2}$$

Using Eqs. S.1 and S.2 produces following equation:

$$f - 1 = \frac{2f_{\text{org}}F_{\text{C,all}} + 2.5F_{\text{SO2}}}{3.5F_{\text{S,all}}} \times \left(\frac{1}{K_{\text{oxy}}} - 1\right). \tag{S.3}$$

Since $F_{\text{C,all}}$, $F_{\text{S,all}}$, F_{SO2}, and f_{org} are not negative, the sign of $(f - 1)$ is identical with that of $(-1 + 1/K_{\text{oxy}})$; therefore,

$$\begin{cases} f > 1 \iff K_{\text{oxy}} < 1 \\ f = 1 \iff K_{\text{oxy}} = 1 \\ f < 1 \iff K_{\text{oxy}} > 1 \end{cases}. \tag{S.4}$$

The oxygenation parameter, K_{oxy}, of a closed gas mixture does not flip from < 1 to > 1 or vice versa as long as reactions occur within the closed

gas mixture [9]. According to the relation between f and $K_{\rm oxy}$ summarized in Eq. S.4, Holland's f number of the closed gas mixture also does not flip from > 1 to < 1 or vice versa as long as reactions occur within the closed gas mixture.

2.2 Temperature dependence of Holland's f number and oxygenation parameter

The temperature dependence of Holland's f number and $K_{\rm oxy}$ is shown in Fig. 9 and 10.

First, assume that the oxygen fugacity of a gas mixture is buffered by that of the ambient melt (i.e., the melt-buffer system in Kadoya et al. [9]). Note that the oxidation state of each case in Fig. 9 is represented by ΔFMQ that is a difference of the logarithm of oxygen fugacity of a gas mixture from that of the fayalite-magnetite-quartz buffer level. For consistency with another case, we represent the oxidation state as $\Delta\mathrm{FMQ}_{2000}$ that is ΔFMQ at 2000 K. However, we assumed that ΔFMQ of the ambient melt, and therefor of the gas mixture, is constant.

For the melt buffer system, a decrease in temperature tends to decrease $K_{\rm oxy}$ (Fig. 9a). Note that Fig. 9a is the same with Fig. 1b of Kadoya et al. [9]; so for detailed information, see Kadoya et al. [9]. On the other hand, the decrease in temperature tends to increase the f number (Fig. 9a).

Here, let us focus on the case of $\Delta\mathrm{FMQ}_{2000} = -0.5$ (dashed lines in Fig. 9). As explained in Kadoya et al. [9], $K_{\rm oxy}$ of $\Delta\mathrm{FMQ}_{2000} = -0.5$ shifts from > 1 to < 1 with a decrease in temperature around 1400 K (Fig. 9a). On the other hand, the f number of $\Delta\mathrm{FMQ}_{2000} = -0.5$ shifts from < 1 to > 1 with a decrease in temperature around 1400 K (Fig. 9b). Both of these shifts indicate that the decrease in temperature results in a shift of atmosphere from oxic to anoxic.

Second, consider a closed gas system. For the closed gas system, a decrease in temperature changes $K_{\rm oxy}$, but $K_{\rm oxy}$ does not flip from < 1 to > 1 or vice versa (Fig. 10a) as explained in Kadoya et al. [9]. Note that Fig. 10a is the same with Fig. 2b of Kadoya et al. [9]. Similarly, the f number does not flip from < 1 to > 1 or vice versa though temperature changes (Fig. 10b). As discussed in Kadoya et al. [9], these results are because of a redox conservation within a closed system.

Thus, temperature dependence of Holland's f number is essentially same with that of $K_{\rm oxy}$ in terms of redox evolution of the atmosphere.

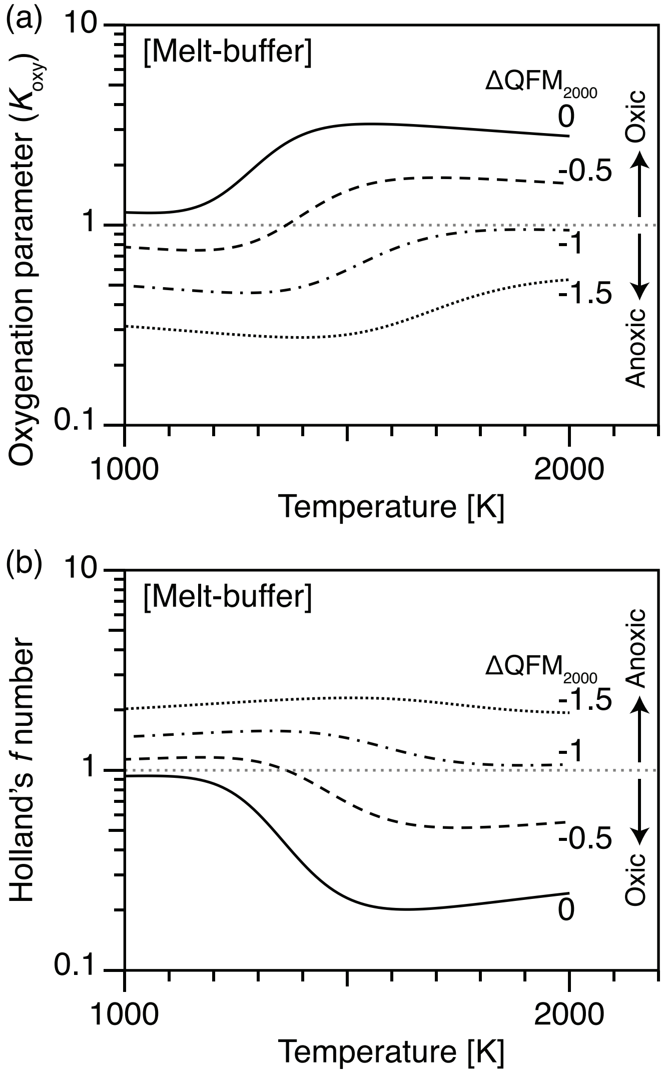

Supplementary Figure 9: Oxygenation effect of a volcanic gas as a function of temperature.: (a) oxygenation parameter (K_{oxy}), and (b) Holland's f number. The melt-buffer system is assumed (see Fig. 1 of Kadoya et al. [9]). Decrease in temperature results in (a) a decrease in K_{oxy} and (b) an increase in f number. In particular, K_{oxy} of $\Delta\mathrm{FMQ}_{2000} = -0.5$ (dashed lines in a) flips from > 1 to < 1 around 1400 K. Similarly, the f number of $\Delta\mathrm{FMQ}_{2000} = -0.5$ (dashed lines in b) flips from < 1 to > 1. Both of these lines show that temperature decrease shift atmosphere from oxic to anoxic if the oxidation state of the melt, and therefore of a volcanic gas, is $\Delta\mathrm{FMQ} = -0.5$. For detailed information, see Kadoya et al. [9].

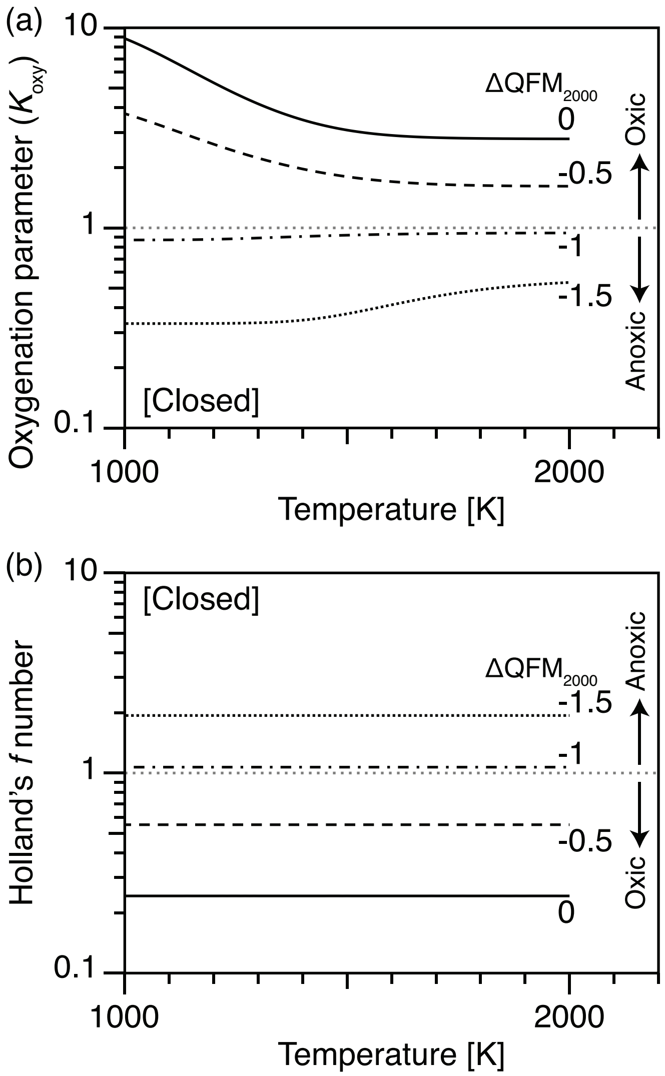

Supplementary Figure 10: Oxygenation effect of a volcanic gas as a function of temperature.: (a) oxygenation parameter ($K_{\rm oxy}$), and (b) Holland's f number. The closed system is assumed (see Fig. 2 of Kadoya et al. [9]). Decrease in temperature changes $K_{\rm oxy}$; however, $K_{\rm oxy}$ does not flip from < 1 to > 1 or vice versa. Similarly, Holland's f number does not flip from < 1 to > 1 or vice versa. For detailed information, see Kadoya et al. [9].

Supplementary Note 3: Oxygen fugacity of mantle

In this study, we model the evolution of the oxygen fugacity of mantle according to the data of Aulbach and Stagno [22] and Nicklas et al. [23]. As shown in Fig. 1a, these two datasets seem to have similar trends but are offset. In this section, we discuss the similarity and difference between the two datasets by using a linear function. Then, we derive the model of the evolution of the oxygen fugacity of mantle.

3.1 Comparison of trends of two datasets of the oxygen fugacity evolution

First of all, we compare trends of the two datasets using an F test. For a null hypothesis, we assumed that the two datasets are fitted by two linear functions whose slopes are the same but whose intercepts are different from each other: i.e., for the data of Aulbach and Stagno [22],

$$\Delta\mathrm{FMQ} = a_1 t + \Delta\mathrm{FMQ}_{1,\mathrm{AS}}, \tag{S.5}$$

and for the data of Nicklas et al. [23],

$$\Delta\mathrm{FMQ} = a_1 t + \Delta\mathrm{FMQ}_{1,\mathrm{N}}. \tag{S.6}$$

Here, t is in the unit of Ga, and the oxygen fugaity is in $\log_{10}$ units relative to the Fayalite-Magnetite-Quartz (FMQ) buffer. The parameters, a_1, $\Delta\mathrm{FMQ}_{1,\mathrm{AS}}$, and $\Delta\mathrm{FMQ}_{1,\mathrm{N}}$, are analytically computed to be -0.29 ± 0.05 [2σ] in the unit of [/Ga], 0.0 ± 0.15 [2σ], and 1.20 ± 0.05 [2σ], respectively. Using these functions, the squared sum of residuals is 96.9.

On the other hand, as an alternative hypothesis, we assumed that the two datasets are fitted by two linear functions whose slopes and intercepts are different from each other: i.e., for the data of Aulbach and Stagno [22].

$$\Delta\mathrm{FMQ} = a_{2,\mathrm{AS}} t + \Delta\mathrm{FMQ}_{2,\mathrm{AS}}, \tag{S.7}$$

and for the dataset of Nicklas et al. [23],

$$\Delta\mathrm{FMQ} = a_{2,\mathrm{N}} t + \Delta\mathrm{FMQ}_{2,\mathrm{N}}. \tag{S.8}$$

Equations S.7 and S.8 are represented by dotted and dashed lines in Fig. 1a, respectively. The parameters for Aulbach and Stagno [22], $a_{2,\mathrm{AS}}$ and $\Delta\mathrm{FMQ}_{2,\mathrm{AS}}$,

are analytically computed to be -0.41 ± 0.17 [2σ] in the unit of [/Ga] and 0.14 ± 0.24 [2σ], respectively. Similarly, the parameters for Nicklas et al. [23], $a_{2,\mathrm{N}}$ and $\Delta\mathrm{FMQ}_{2,\mathrm{N}}$, are analytically computed to be -0.27 ± 0.05 [2σ] in the unit of [/Ga] and 1.2 ± 0.1 [2σ], respectively. Using these functions, the squared sum of residuals is 94.7.

The number of data is 12 for Aulbach and Stagno [22] and 18 for Nicklas et al. [23]. We use four parameters for the null hypothesis and three parameters for the alternative hypothesis. So, the F-value is calculated as follows:

$$F = \left(\frac{96.9 - 94.7}{(12 + 18 - 3) - (12 + 18 - 4)} \right) \left(\frac{94.7}{12 + 18 - 4} \right)^{-1} \sim 0.61. \tag{S.9}$$

Considering an F distribution with this F-value (0.61), the p-value is calculated to be 44%. Since the p-value is higher than a canonical rejection rate (5%), we cannot reject the null hypothesis. Hence, we consider that the slopes of the fitting functions for the two datasets are the same as each other.

3.2 Comparison of offsets of two datasets of the oxygen fugacity evolution

As explained above, we fit the two datasets by linear functions whose slopes are the same as each other. Then, we compare the intercepts of the linear functions using the F test.

For a null hypothesis, we assumed that the two datasets are fitted by one linear function. The fitting function for both of Aulbach and Stagno [22] and Nicklas et al. [23] is as follows:

$$\Delta\mathrm{FMQ} = a_3 t + \Delta\mathrm{FMQ}_3. \tag{S.10}$$

The parameters, a_3 and $\Delta\mathrm{FMQ}_3$, are analytically computed to be -0.21 ± 0.05 [2σ] in the unit of [/Ga] and 0.9 ± 0.1 [2σ], respectively. Using this function, the squared sum of residuals is 329.3.

On the other hand, for an alternative hypothesis, we assumed that that the two datasets are fitted by two linear functions whose slopes are the same but intercepts are different from each other: i.e., the null hypothesis above (see Eq. S.5 and S.6, and related explanation). As explained above, using Eq. S.5 and S.6, the squared sum of residuals is 96.9.

The number of data is 12 for Aulbach and Stagno [22] and 18 for Nicklas et al. [23], and we use three parameters for the null hypothesis and 2 parameters

for the alternative hypothesis. So, the F-value is calculated as follows:

$$F = \left(\frac{329.3 - 96.9}{(12+18-2)-(12+18-3)}\right)\left(\frac{96.9}{12+18-3}\right)^{-1} \sim 64.8. \quad \text{(S.11)}$$

Considering an F distribution with this F-value (64.8), the p-value is calculated to be 1.2×10^{-6}%. Since the p-value is lower than the canonical rejection rate (5%), we reject the null hypothesis.

3.3 Oxygen fugacity of the modern mantle

As explained above, the two datasets of the oxygen fugacity (f_{O2}) evolutions [22, 23] are fitted by linear functions whose slopes are the same but whose intercepts are different. The difference in the absolute values of f_{O2} is due to the issue of the inter-calibration of different oxybarometers, or due to the difference in mantle melting process between ridge and plume [24, 23].

In this study, we anchor the evolution of the f_{O2} at the value which is evaluated using the oxidation state of iron in the modern mid ocean ridge (MORB) samples [19]. According to O'Neill et al. [19], the f_{O2} of the modern MORB is 0.2 ± 0.6 [2σ] in $\log_{10}$ units relative to the FMQ buffer. So, for a standard case of this study, we assumed that the oxygen fugacity of the modern mantle is FMQ+0.2. The variation in the modern oxygen fugacity is considered later as a parameter study.

Setting the oxygen fugacity of the modern mantle, we can regard the intercepts calculated above (i.e., $\Delta\mathrm{FMQ}_{1,\mathrm{AS}}$ in Eq. S.5, and $\Delta\mathrm{FMQ}_{1,\mathrm{N}}$ in Eq. S.6) as the offset from the evolution of the oxygen fugacity. For the standard case, the average offset is +0.2 for Aulbach and Stagno [22], and -1.00 for Nicklas et al. [23].

Supplementary References

[1] Krissansen-Totton, J., Buick, R., & Catling, D. C. A statistical analysis of the carbon isotope record from the Archean to Phanerozoic and implications for the rise of oxygen. *Am. J. Sci.* **315,** 275–316 (2015).

[2] Herzberg, C., Condie, K., & Korenaga, J. Thermal history of the Earth and its petrological expression. *Earth Planet. Sc. Lett.* **292,** 79–88 (2010).

[3] Holland, H. D. *The Chemical Evolution of the Atmosphere and Oceans.* (Princeton University Press, Princeton, 1984).

[4] Nisbet, E. G., Cheadle, M. J., Arndt, N. T., & Bickle, M. J. Constraining the potential temperature of the Archean mantle - a review of the evidence from komatiites. *Lithos* **30,** 291–307 (1993).

[5] Aulbach, S. & Arndt, N. T. Eclogites as palaeodynamic archives: Evidence for warm (not hot) and depleted (but heterogeneous) Archaean ambient mantle. *Earth Planet. Sc. Lett.* **505,** 162–172 (2019).

[6] Wones, D. R. & Gilbert, M. C. The fayalite-magnetite-quartz assemblage between 600 and 800 c. *Am. J. Sci.*, 267:480–488, 1969.

[7] Till, C. B., Grove, T. L., & Withers, A. C. The beginnings of hydrous mantle wedge melting. *Contrib. Mineral. Petr.* **163,** 669–688 (2012).

[8] Carmichael, I. S. E. The redox states of basic and silicic magmas: a reflection of their source regions? *Contrib. Mineral. Petr.* **106,** 129–141 (1991).

[9] Kadoya, S., Catling, D. C., Nicklas, R. W., Puchtel, I. S., & Anbar, A. D. Mantle cooling causes more reducing volcanic gases and gradual reduction of the atmosphere. *Geochem. Perspec. Lett.* **13,** 25–29 (2020).

[10] Moussallam, Y., Oppenheimer, C., & Scaillet, B. On the relationship between oxidation state and temperature of volcanic gas emissions. *Earth Planet. Sc. Lett.* **520,** 260–267 (2019).

[11] Kump, L. R. & Barley, M. E. Increased subaerial volcanism and the rise of atmospheric oxygen 2.5 billion years ago. *Nature* **448,** 1033–1036 (2007).

[12] Gaillard, F., Scaillet, B., & Arndt, N. T. Atmospheric oxygenation caused by a change in volcanic degassing pressure. *Nature* **478,** 229–233 (2011).

[13] Brounce, M., Stolper, E., & Eiler, J. Redox variations in mauna kea lavas, the oxygen fugacity of the hawaiian plume, and the role of volcanic gases in Earth's oxygenation. *P. Natl. Acad. Sci. USA* **114,** 8997–9002 (2017).

[14] Holland, H. D. Why the atmosphere became oxygenated: A proposal. *Geochim. Cosmochim. Ac.* **73,** 5241–5255 (2009).

[15] Sleep, N. H. Dioxygen over geologic time. in *Metal Ions in Biological Systems, Vol. 43, Biogeochemical Cycles of Elements* (eds. Sigel, H. & Sigel, R.) 49–73 (Taylor & Francis, Boca Raton, FL 2005).

[16] Canfield, D. E., Rosing, M. T., & Bjerrum, C. Early anaerobic metabolisms. *Philos. T. R. Soc. B.* **361,** 1819–1834 (2006).

[17] Kasting, J. F. What caused the rise of atmospheric O2? *Chem. Geol.* **362,** 13–25 (2013).

[18] Sleep, N. H. Evolution of the Earth: Plate tectonics through time. in*Treatise on Geophysics (Second Edition)* (eds. Schubert, G.) 145–172 (Elsevier, Oxford, 2015).

[19] O'Neill, H. S. C., Berry, A. J., & Mallmann, G. The oxidation state of iron in mid-ocean ridge basaltic (MORB) glasses: Implications for their petrogenesis and oxygen fugacities. *Earth Planet. Sc. Lett.* **504,** 152–162 (2018).

[20] Holland, H. D. Volcanic gases, black smokers, and the Great Oxidation Event. *Geochim. Cosmochim. Ac.* **66,** 3811–3826 (2002).

[21] Catling, D. C. & Claire, M. W. How Earth's atmosphere evolved to an oxic state: A status report. *Earth Planet. Sc. Lett.* **237,** 1–20 (2005).

[22] Aulbach, S. & Stagno, V. Evidence for a reducing Archean ambient mantle and its effects on the carbon cycle. *Geology* **44,** 751–754 (2016).

[23] Nicklas, R. W. et al. Secular mantle oxidation across the Archean-Proterozoic boundary: Evidence from V partitioning in komatiites and picrites. *Geochim. Cosmochim. Ac.* **250,** 49–75 (2019).

[24] Mallmann, G. & O'Neill, H. S. Calibration of an empirical thermometer and oxybarometer based on the partitioning of Sc, Y and V between olivine and silicate melt. *J. Petrol.* **54,** 933–949 (2013).
